# Corticosteroid plus β_2_-agonist in a single inhaler as reliever therapy in intermittent and mild asthma: a proof-of-concept systematic review and meta-analysis

**DOI:** 10.1186/s12931-017-0687-6

**Published:** 2017-12-06

**Authors:** Gang Wang, Xin Zhang, Hong Ping Zhang, Lei Wang, De Ying Kang, Peter J. Barnes, Gang Wang

**Affiliations:** 1 Pneumology Group, Department of Integrated Traditional Chinese and Western Medicine, State Key Laboratory of Biotherapy, West China Hospital, Sichuan University, and Collaborative Innovation Center for Biotherapy, Chengdu, 610041 People’s Republic of China; 2 Pneumology Group, Department of Integrated Traditional Chinese and Western Medicine, West China Hospital, Sichuan University, Chengdu, 610041 People’s Republic of China; 3 West China School of Medicine, Sichuan University, Chengdu, 610041 People’s Republic of China; 4 Department of Evidence-based Medicine and Clinical Epidemiology, West China Hospital, Sichuan University, Chengdu, China; 5 National Heart & Lung Institute, Imperial College, London, UK

**Keywords:** Combination of corticosteroid and fast-onset-acting β_2_-agonist, Inhaled corticosteroids, Short-acting β_2_-agonist, Meta-analysis

## Abstract

**Background:**

Current guidelines recommend a single inhaler maintenance and reliever therapy (SMART) regimen for moderate to severe asthma. However, evidence for the inhaled corticosteroid plus fast-onset-acting β_2_-agonist (ICS/FABA) as reliever therapy in management of intermittent and mild asthma patients is lacking.

**Objective:**

To systematically explore efficacy and safety of the proof-of-concept of the ICS plus FABA regimen in a single inhaler as reliever therapy across children and adults with intermittent and mild persistent asthma.

**Methods:**

We searched online bibliographic databases for randomized controlled trials (RCTs) involving the as-needed use of ICS/FABA as monotherapy in intermittent or mild asthma patients. The primary outcomes were exacerbations and the hazard ratio (HR) of the time to first exacerbation.

**Results:**

Six RCTs (*n* = 1300) met the inclusion criteria. Compared with the as-needed FABA regimen, the as-needed use of ICS/FABA as monotherapy statistically reduced exacerbations (RR = 0.56, *P* = 0.001). Compared with regular ICS regimen, the as-needed ICS/FABA therapy had slightly higher risk of exacerbations (RR = 1.39, *P* = 0.011). The HR for time to first exacerbations in the ICS/FABA regimen was significant lower when compared with FABA regimen (HR = 0.52, *P* = 0.002) but had no difference when compared with ICS regimen (HR = 1.30, *P* = 0.286). The corticosteroid exposure in the daily ICS regimen was 2- to 5-fold compared with as-needed use of ICS/FABA regimen.

**Conclusions:**

Our analysis shows that the ICS/FABA as a symptom-driven therapy may be a promising alternative regimen for the patients with intermittent or mild asthma, but it needs further real-world RCTs to confirm these findings.

**Electronic supplementary material:**

The online version of this article (10.1186/s12931-017-0687-6) contains supplementary material, which is available to authorized users.

## Introduction

Asthma is characterized by airway inflammation, airway hyper-responsiveness, and variable airflow limitation [1], with an estimated 300 million affected individuals in the world [2, 3]. Currently, clinical research and management initiatives primarily focus on severe asthma [4–6], while more than half of patients have intermittent or mild diseases [7–9], and there is a largely unexplored but important burden of disease in this group [10]. Short-acting β_2_-agonists (SABA) can quickly relieve the symptoms [11], but it has no significant anti-inflammatory effects [12].Intermittent or mild asthma patients are recommended to initiate treatment with maintenance of low-dose inhaled corticosteroids (ICS) if they require a SABA more than twice a week [13, 14] or twice a month [1]. In addition, in a real-life setting, poor adherence of ICS as a controller is associated with significant asthma-related morbidity. Furthermore, some patients with intermittent or mild asthma prefer to take anti-asthma therapy intermittently and occasionally when they experience few symptoms [15, 16]. Therefore, alternative strategies for long-term management of intermittent or mild asthma is to be needed.

It is now well established that a fixed combination of ICS/LABA inhaler for both maintenance and reliever therapy (SMART regimen), which significantly reduces the risk of severe exacerbations and systemic corticosteroid exposure compared with standard fixed-dose regimen in moderate and severe asthma patients, has been recommended for patients with steps 3 to 5 in guidelines [1]. However, it could not be generalized to patients with steps 1 and 2 being equivalent to intermittent or mild persistent asthma (GINA steps 1 and 2) (Table 1), because there is a lack of evidence for the combination corticosteroid/fast-onset-acting β_2_-agonist (ICS/FABA) in a single inhaler as reliever therapy in the management of these patients. A proof-of-concept study indicated that in patients with mild asthma, the symptom-driven use of ICS and SABA in a single inhaler resulted in efficacy similar to that seen with regular ICS therapy [17]. Hence, in this proof-of-concept systematic review, we systematically explored the efficacy and safety of the ICS plus FABA regimen in a single inhaler as reliever therapy compared with the as-needed use of FABA regimen and the daily use of ICS regimen in children and adults with intermittent and mild persistent asthma based on randomized controlled trials.

**Table 1:** Recommendations of step1/2 treatments in different guidelines

Guidelines	Step 1	Step 2
GINA [1]	Preferred controller choice, none;	Preferred controller choice, low dose ICS;
	Other controller option, low dose ICS;	Other controller option, LTRA, theophylline;
	Reliever, as-needed SABA.	Reliever, as-needed SABA.
NAEPP [13]	Preferred controller choice, none;	Preferred controller choice, low dose ICS;
	Other controller option, none;	Other controller option, cromolyn, LTRA, nedocromil, theophylline;
	Reliever, as-needed SABA.	Reliever, as-needed SABA.
British Guideline on the Management of Asthma [53]	Preferred controller choice, none;	Preferred controller choice, low dose ICS;
	Other controller option, none;	Other controller option, chromones, LTRA, theophylline;
	Reliever, as-needed SABA, inhaled ipratropium bromide or theophylline.	Reliever, as-needed SABA.
The Chinese guideline for Asthma Management and Prevention [54]	Preferred controller choice, none;	Preferred controller choice, low dose ICS;
	Other controller option, low dose ICS;	Other controller option, LTRA, ICS/LABA;
	Reliever, as-needed SABA.	Reliever, as-needed SABA.
Spanish guideline on the management of asthma [55]	Preferred controller choice, none;	Preferred controller choice, low dose ICS, LTRA;
	Other controller option, none;	Other controller option, chromones, theophylline;
	Reliever, as-needed SABA, inhaled anticholinergic.	Reliever, as-needed SABA.
Japanese guidelines for adult asthma [56]	Preferred controller choice, low dose ICS;	Preferred controller choice, ICS/LABA;
	Other controller option, LTRA, theophylline;	Other controller option, low to medium dose ICS, ICS and LTRA, ICS and theophylline;
	Reliever, as-needed SABA.	Reliever, as-needed SABA.
The Saudi Initiative for Asthma [57]	Preferred controller choice, none;	Preferred controller choice, low dose ICS;
	Other controller option, none;	Other controller option, LTRA;
	Reliever, as-needed SABA.	Reliever, as-needed SABA.

*GINA* the Global Initiative for Asthma, *NAEPP* the National Asthma Education and Prevention Program, *ICS* inhaled corticosteroids, *LTRA* Leukotriene receptor antagonists, *SABA* short-acting β_2_-agonist, *ICS/LABA* inhaled corticosteroids/long-acting β_2_-agonist

## Methods

This study was in adherence to the Preferred Reporting in Systematic Reviews and Meta-Analyses (PRISMA) guidelines [18].

### Selection criteria

The eligible studies were randomized controlled trials (RCTs) including patients with intermittent or mild persistent asthma. The eligible interventions included the as-needed use of ICS plus FABA regimen as only one treatment in a single inhaler or separate inhalers in comparison with the regular ICS regimen or the as-needed use of FABA regimen. Any types of fast-onset-acting β_2_-agonists such as SABA (salbutamol, terbutaline or others) or FABA (formoterol but not salmeterol) were allowed.

### Data sources and searching

We searched MEDLINE (Ovid), EMBASE (Ovid), Epub Ahead of Print, In-Process & Other Non-Indexed Citations (Ovid) and Cochrane Central Register of Controlled Trials (CENTRAL, Ovid) up to October 10, 2017, for randomized controlled trials involving the as-needed use of ICS/FABA as monotherapy in intermittent or mild asthma patients. To increase sensitivity for founding the intermittent and mild asthma trials, we included broader asthma severity terms contained total asthma spectrum (The medical subject headings (MeSH) terms used as described in Additional file 1: Table S1). There was no language restriction for the search. We also manually reviewed reference lists of relevant reports and contacted with the manufacturer of budesonide/formoterol inhaler (Symbicort®, AstraZeneca AB) for any unpublished studies and/or additional unpublished data from published studies. To identify ongoing trials, we also searched the WHO International Clinical Trials Registry Platform (ICTRP) and ClinicalTrials.gov.

### Study selection

To validate this proof-of-concept of the combination as-needed use of the ICS plus FABA regimen in intermittent and mild asthma, we included all studies that involved the use of the ICS and the FABA in a single inhaler or separate inhalers as reliever therapy. Two reviewers (XZ & GW) independently selected articles on the basis of title and/or abstract for full text scrutiny. Disagreements were resolved by consensus or, if required, a third reviewer serving as the arbitrator (GW as the corresponding author).

### Data extraction

Two reviewers independently extracted information from included studies for the following characteristics such as authors, study design, total duration of study, details of any ‘run in’ period, study centers and location, inclusion criteria, exclusion criteria, diagnostic criteria of asthma, asthma severity, sample size, age, gender, baseline lung function, inhaler device, daily dose of steroid presented as beclomethasone dipropionate (BDP) equivalent, adherence, dropouts or withdrawals and outcomes.

### Quality assessment

The bias risk of the different studies was assessed with the components recommended by the Cochrane Collaboration for randomized trials [19]. These components include random sequence generation, allocation concealment, blinding of participants and personnel, blinding of outcome assessment, incomplete outcome data, selective reporting and other bias. For each component, individual team members judged whether the risk of bias in a given study was “low,” “high,” or “unclear.” Any disagreements were referred to the third reviewer.

### Primary and secondary outcomes

The primary objective of this study was to explore the efficacy of the as-needed use ICS/FABA regimen, and therefore the primary outcome was the exacerbations defined by the criteria of moderate to severe exacerbations of American Thoracic Society (ATS)/European Respiratory Society (ERS) [20]. Furthermore, we also calculated the severe exacerbations and the hazard ratio (HR) of the time to first exacerbation after randomization within these included studies.

The secondary outcomes included nocturnal awakenings times, the percentage of asthma symptom-free days, the number of rescue medication required per day, forced expiratory volume in one second (FEV_1_) percentage of predicted value. In addition, in terms of the safety profile, we assessed the number of dropout, serious adverse events, corticosteroid exposure and linear growth during the study period just in the children and adolescent subgroup.

### Statistical analysis

We treated exacerbations data using patients as the unit of analysis (rather than events) to avoid repeating. Where zero counts existed for an dichotomous outcome in one arm of a trial, we added a value of 0.5 to permit meta-analysis, and where zero counts existed in both arms of a trial, we omitted the trial from the analysis of that outcome according to Cochrane principles [19]. We presented dichotomous data as risk ratio (RR), continuous data as the standardized mean difference (SMD) and time-to-event data as hazard ratio (HR) with 95% confidence intervals. Specially, a software named GetData Graph Digitizer v.2.26 [21] (DR MyCommerce, Inc.) was used to dig out the detailed data from Kaplan–Meier curves of the time to first exacerbation for calculating the HR, which was described in detail in the Methods section in this article’s Additional file 1. For missing data, we contacted investigators or study sponsors in order to obtain where possible. Otherwise, we dealt with missing data according to the Cochrane handbook recommending principals [19, 22]. All analyses were performed using an intention to treat approach. For the primary outcome of exacerbations, we calculated the number needed to treat (NNT) for assessing the different levels of risk. Heterogeneity was assessed with the Q statistic and the I^2^ statistic. A random-effects model [23] was used to pool data if substantial heterogeneity was observed (I^2^ > 50% or *P* < 0.1 for Q statistic), otherwise we used a fixed-effects model [24]. If there were more than 10 trials, a funnel plot would be created to explore possible publication bias. The quality of a body of evidence for primary outcomes was rated using the Grading of Recommendations, Assessment, Development and Evaluation (GRADE) system [25] (GRADEpro Guideline Development Tool, McMaster University, 2015).

In addition, because adherence is very important in regular or fixed maintenance therapy, we undertook an additional meta-analysis of auxiliary information was performed to pool real-life adherence of the daily use of ICS treatment in patients with persistent asthma who were present in a real-world setting. The detailed method and results were provided in the Methods and Results section in this article’s Additional file 1.

Data were combined with the Stata 14.0 software (College Station, TX). Statistical significance was assumed for *P* < 0.05.

## Results

### Studies retrieved and characteristics

**Fig. 1:**
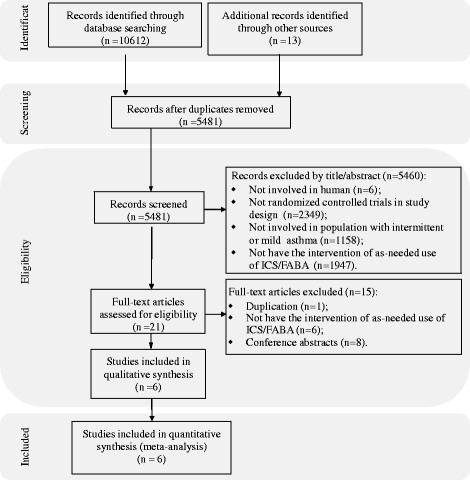
Flow of study identification, inclusion, and exclusion

Figure 1 shows details of study identification, inclusion, and exclusion. Our search strategy initially yielded 10,612 citations, and the AstraZeneca (AstraZeneca AB) provided thirteen studies. Finally, six RCTs were included in this meta-analysis [17, 26–30].

Table 2 shows the characteristics of the included studies. Generally, there were five parallel trials and one crossover trial [26]. According to the recommendation of the Cochrane Handbook for Systematic Reviews of Interventions [19], we judged the suitability and acceptability of the cross-over design in our study (Additional file 1: Table S2). One thousand and three hundred subjects were included for analysis (Table 2). There were 674 (6 studies; 46.2% female; median age: 25.9 [range: 2.3–39.9] years), 317 (5 studies; 54.9% female; median age: 23.6 [range: 2.3–40.6] years) and 609 (5 studies; 46.1% female; median age: 23.4 [range: 2.4–39.9] years) subjects in the as-needed use of ICS/FABA regimen, the as-needed use of FABA regimen and in the daily use of ICS regimen, respectively. The diagnostic criteria for asthma in the included studies were based on the guidelines recommendation except for the study by Papi et al. [30] on frequent wheezing in the previous 6 months. There was one study on intermittent asthma and 5 studies on mild persistent asthma. All subjects in these included studies were requested to have the adherence of daily ICS with more than 75%. The average FEV_1_ percentage of predicted value were 95.4 (SD = 13.1), 94.8 (SD = 12.0) and 94.0 (SD = 12.1) for the as-needed use of ICS/FABA, as-needed use of FABA and the daily ICS regimens groups, respectively. The ICS/FABA regimens used included beclomethasone/salbutamol, budesonide/formoterol, beclomethasone and salbutamol, beclomethasone/salbutamol and fluticasone propionate and salbutamol, respectively. The regular ICS regimens used involved beclomethasone, budesonide and fluticasone propionate, respectively. The FABA regimens used were presented as formoterol, terbutaline and salbutamol. The median intervention duration was 24 (ranged from 6 to 44) weeks.

**Table 2:** Characteristics of included studies

Variables	Haahtela 2006 [27]	Papi 2007 [17]	Papi 2009 [30]	Martinez 2011 [29]	Lazarinis 2014 [28]	Fitzpatrick 2016 [26]
Countries	Finland and Sweden	Multinational	Italy	USA	Sweden and Norway	USA
Study design	Multicenter, randomized, double-blind, parallel trial	Multicenter, randomized, double-blind, parallel trial	Multicenter, randomized, double-blind, parallel trial	Multicenter, randomized, double-blind, parallel trial	Randomized, double-blind, parallel trial	Multicenter, randomized, double-blind, crossover trial
Duration (weeks)	24	24	12	44	6	16
Diagnostic criteria of asthma	GINA	The EPR-2 guideline	Frequent wheezing^a^	The EPR-3 guideline	GINA	The EPR-3 guideline
Asthma severity	Intermittent	Mild	Mild	Mild	Mild	Mild
Intervention	ICS/FABA regimen, budesonide/formoterol, 1 inhalation, 160/4.5 μg each as-needed; FABA regimen, formoterol, 1 inhalation, 4.5 μg, as-needed	ICS/FABA regimen, beclomethasone/salbutamol, 1 inhalation, 250/100 μg each, as-needed; FABA regimen, salbutamol, 1 inhalation, 100 μg each, as-needed; ICS regimen, beclomethasone, 1 inhalation, 250 μg each, twice daily	ICS/FABA regimen, beclomethasone/salbutamol, 1 vial nebulization, 800/1600 μg each, as-needed; FABA regimen, salbutamol, 1 vial nebulization, 2500 μg each, as-needed ICS, beclomethasone, 1 vial nebulization, 400 μg each, twice daily	ICS regimen, beclomethasone (HFA), 2 inhalations, 40 μg each, twice daily or as-needed; FABA regimen, salbutamol, 2 inhalations, 90 μg each, as-needed	ICS/FABA regimen, budesonide/formoterol, 1 inhalation, 200/6 μg each, as-needed; FABA regimen, terbutaline, 1 inhalation, 500 μg each, as-needed; ICS regimen, budesonide, 1 inhalation, 400 μg each, once daily	ICS regimen, fluticasone propionate, 2 inhalations, 44 μg each, twice daily or as-needed; FABA regimen, salbutamol, 2 inhalations, 90 μg each, as-needed
Adherence of daily ICS	NA	91.62%	99.4%	85%	98.25%	96%
Beclomethasone equivalent consumption (μg/day, Mean ± SD)						
ICS/FABA	162.8	110 ± 150.3	179.8 ± 256	30	203.75 ± 100	77.14
ICS	–	458.1 ± 103.3	795.2 ± 81	170	491.25 ± 15	342.86
Number of patients						
ICS/FABA	45	122	110	74	23	NA
FABA	47	118	56	74	22	NA
ICS	–	106	110	72	21	NA
Total	92	346	276	220	66	300
Age (years, mean ± SD)						
ICS/FABA	34.8 ± 19.9	36.8 ± 13.1	2.26 ± 0.79	10.4 ± 2.8	31 ± 12	NA
FABA	36.5 ± 12.1	40.6 ± 13.8	2.29 ± 0.78	10.4 ± 3.2	28 ± 12	NA
ICS	–	37.9 ± 13.5	2.35 ± 0.81	10.8 ± 3.5	26 ± 10	NA
Total	NA	NA	NA	NA	NA	3.325 ± 1.1
Sex (% female)						
ICS/FABA	66	59	38.2	48	39	NA
FABA	72	58.5	39.3	45	72	NA
ICS	–	57.5	41.8	42	52	NA
Total	NA	NA	NA	NA	NA	40.3
Number of dropout						
ICS/FABA	1	22	4	13	1	18^b^
FABA	5	18	3	24	0	–
ICS	–	17	2	9	3	12^b^

*ICS* inhaled corticosteroids, *FABA* fast-onset-acting β_2_-agonist, *ICS/FABA* inhaled corticosteroids/fast-onset-acting β_2_-agonist

^a^Frequent wheezing defined as a documented history of at least three episodes of wheezing requiring medical attention in the previous 6 months

^b^Treatment failure criteria were met in a single 16-week treatment arm

The quality of reporting in the reviewed studies was generally good. The risk of bias is shown in Additional file 1: Table S2. All studies were multicenter trials except Lazarinis et al’s study [28]. All the included studies were randomized double-blind trials, except the study of Papi et al. [30] was an unclear risk in random sequence generation, Lazarinis et al’s study [28] there was an unclear risk in blinding and Haahtela et al’s study [27] there was an unclear risk in allocation concealment and blinding.

### Primary outcomes

**Fig. 2:**
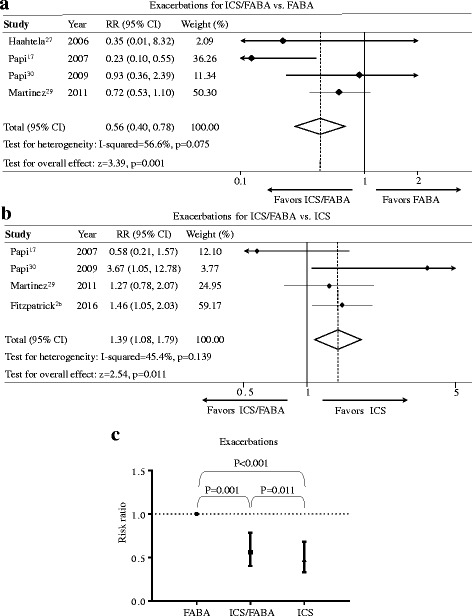
Effects of the as-needed ICS/FABA, daily ICS, and as-needed FABA regimens on moderate to severe exacerbations. **a** ICS/FABA vs FABA regimen; **b** ICS/FABA vs ICS regimen; **c** Risk ratio across three regimens. ICS, inhaled corticosteroids; FABA, fast-onset-acting β_2_-agonist; ICS/FABA, inhaled corticosteroids/fast-onset-acting β_2_-agonist

Compared with the as-needed FABA regimen, the as-needed use of ICS/FABA as monotherapy statistically reduced moderate to severe exacerbations (RR = 0.56, 95%-CI = [0.40, 0.78], *P* = 0.001, I^2^ = 56.6%, Fig. 2a, Table 3). The number needed to treat for an additional beneficial outcome (NNTB) was 10 and the number of avoided events per 1000 was 101 (95%-CI = [51, 138]). Compared with regular ICS regimen, the as-needed ICS/FABA regimen had slightly higher risk of moderate to severe exacerbations (RR = 1.39, 95%-CI = [1.08, 1.79], *P* = 0.011, I^2^ = 45.4%, Fig. 2b), and the number needed to treat for an additional harmful outcome (NNTH) was 17 and the number of excess events per 1000 was 57 (95%-CI = [12, 116]).

**Table 3:** Subgroup analysis of exacerbations

Comparisons	Exacerbations						Severe exacerbations					
	No. of Studies	ICS/FABA	FABA or ICS	RR (95% CI)	*P* value	Power	No. of Studies	ICS/FABA	FABA or ICS	RR (95% CI)	*P* value	Power
ICS/FABA vs. FABA regimen												
All	4 [17, 27, 29, 30]	42/348	68/295	0.56 (0.40, 0.78)	0.001	1.00	3 [17, 27, 29]	25/238	41/239	0.64 (0.44, 0.95)	0.026	0.89
Adult	2 [17, 27]	6/167	26/165	0.24 (0.10, 0.54)	0.001	0.98	2 [17, 27]	0/167	5/165	0.17 (0.02, 1.35)	0.093	0.47
Children and adolescent	2 [29, 30]	36/181	42/130	0.76 (0.53, 1.10)	0.147	0.76	1 [29]	25/71	36/74	0.72 (0.49, 1.07)	0.107	0.34
ICS/FABA vs. ICS regimen												
All	4 [17, 26, 29, 30]	111/553	79/537	1.39 (1.08, 1.79)	0.011	1.00	3 [17, 26, 29]	94/443	70/427	1.34 (1.02,1.75)	0.034	0.91
Adult	1 [17]	6/122	9/106	0.58 (0.21, 1.57)	0.284	0.17	1 [17]	0/122	3/106	0.12 (0.01,2.38)	0.166	0.22
Children and adolescent	3 [26, 29, 30]	105/431	70/431	1.50 (1.15, 1.96)	0.003	1.00	2 [26, 29]	94/321	67/321	1.40 (1.07,1.84)	0.014	0.81

*no.* number, *CI* confidence interval, *ICS* inhaled corticosteroids, *FABA* fast-onset-acting β_2_-agonist, *ICS/FABA* inhaled corticosteroids/fast-onset-acting β_2_-agonist

**Fig. 3:**
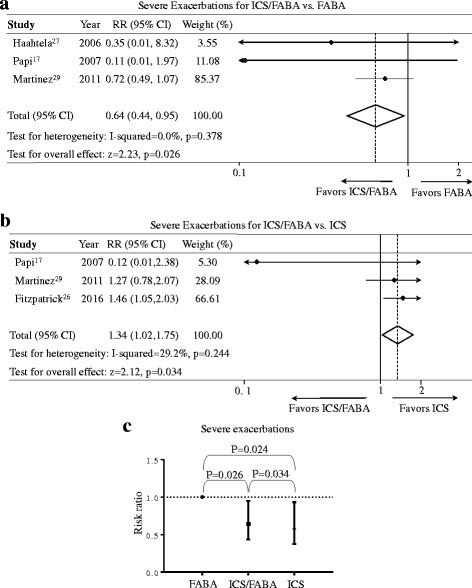
Effects of the as-needed ICS/FABA, daily ICS, and as-needed FABA regimens on severe exacerbations. **a** ICS/FABA vs FABA regimen; **b** ICS/FABA vs ICS regimen; **c** Risk ratio across three regimens. ICS, inhaled corticosteroids; FABA, fast-onset-acting β_2_-agonist; ICS/FABA, inhaled corticosteroids/fast-onset-acting β_2_-agonist

We also explored the difference of severe exacerbations [20] between the as-needed ICS/FABA, the regular ICS and the as-needed FABA regimens (Fig. 3). As a result, the as-needed ICS/FABA regimen significantly reduced severe exacerbations (RR = 0.64, 95%-CI = [0.44, 0.95], *P* = 0.026, I^2^ = 0.0%) compared to FABA regimen and the NNTB was 16 and the number of avoided events per 1000 was 62 (95%-CI = [9, 96]). In comparison with the regular ICS regimen, the as-needed ICS/FABA regimen had increased risk of severe exacerbations (RR = 1.34, 95%-CI = [1.02, 1.75], *P* = 0.034, I^2^ = 29.2%) and the NNTH was 18 and the number of excess events per 1000 was 56 (95%-CI = [3, 123]).

**Fig. 4:**
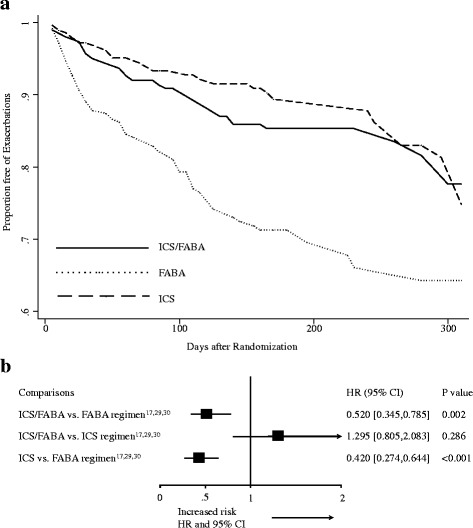
Life-table curves of the time to first exacerbation (**a**) and hazard ratio (**b**) across the as-needed ICS/FABA (*n* = 303), as-needed FABA (*n* = 248) and regular ICS (*n* = 288) regimens. ICS, daily use of inhaled corticosteroids regimen; FABA, as-needed use of fast-onset-acting β_2_-agonist regimen; ICS/FABA, as-needed use of inhaled corticosteroids/fast-onset-acting β_2_-agonist regimen

Like the study by Yancey and colleagues [31], we grouped the data of the time to first exacerbation, obtained by GetData Graph Digitizer [32], into a five-day interval life table and established a life-table curves to illustrate the difference in the time to first exacerbation for each of the three treatments (Fig. 4a). As a result, the hazard ratio (HR) for time to first exacerbations after randomization in the ICS/FABA regimen was significant lower when compared with FABA regimen (HR = 0.520, 95%-CI = [0.345, 0.785], *P* = 0.002) but did not reach statistical difference when compared with ICS regimen (HR = 1.295, 95%-CI = [0.805, 2.083], *P* = 0.286) (Fig. 4b).

The quality of evidence body rated by GRADE for primary outcomes is summarized in Table 4. Compared with the as-needed FABA regimen, the as-needed use of ICS/FABA as monotherapy probably reduces moderate to severe exacerbations and the hazard for the time to first exacerbation (moderate-quality evidence). The as needed ICS/FABA regimen probably has slightly higher risk of moderate to severe exacerbations and increases the hazard for the time to first exacerbation compared with regular ICS regimen (moderate-quality evidence). In addition, compared with FABA regimen, regular ICS regimen probably reduces the hazard for the time to first exacerbation (moderate-quality evidence).

**Table 4:** The quality of evidence assessment for as-needed use of ICS/FABA in intermittent and mild asthma by GRADE approach

Outcome No. of participants (studies)	Relative effect (95% CI)	Anticipated absolute effects (95% CI)			Certainty	What happens
		Comparison group	Intervention group	Difference		
ICS/FABA compared to FABA for intermittent or mild persistent asthma						
Patient or population: intermittent or mild persistent asthma Setting: inpatients and outpatient Intervention: ICS/FABA Comparison: FABA						
Exacerbations follow up: range 12 weeks to 44 weeks No. of participants: 643 (4 RCTs) [17, 27, 29, 30]	RR 0.56 (0.40 to 0.78)	23.1%	12.9% (9.2 to 18.0)	10.1% fewer (13.8 fewer to 5.1 fewer)	MODERATE^a^	Compared with the as-needed FABA regimen, the as-needed use of ICS/FABA as monotherapy probably reduces moderate to severe exacerbations
Severe exacerbations follow up: range 24 weeks to 44 weeks No. of participants: 477 (3 RCTs) [17, 27, 29]	RR 0.64 (0.44 to 0.95)	17.2%	11.0% (7.5 to 16.3)	6.2% fewer (9.6 fewer to 0.9 fewer)	MODERATE^a^	Compared with the as-needed FABA regimen, the as-needed use of ICS/FABA as monotherapy probably reduces severe exacerbations
Time to first exacerbation follow up: range 12 weeks to 44 weeks No. of participants: 551 (3 RCTs) [17, 29, 30]	HR 0.520 (0.345 to 0.785)	27.0%	15.1% (10.3 to 21.9)	11.9% fewer (16.7 fewer to 5.1 fewer)	MODERATE^a^	Compared with FABA regimen, ICS/FABA regimen probably reduces the HR for the time to first exacerbation
ICS/FABA compared to ICS for intermittent or mid persistent asthma						
Patient or population: intermittent or mid persistent asthma Setting: inpatients and outpatient Intervention: ICS/FABA Comparison: ICS						
Exacerbations follow up: range 12 weeks to 44 weeks No. of participants: 1090 (4 RCTs) [17, 26, 29, 30]	RR 1.39 (1.08 to 1.79)	14.7%	20.4% (15.9 to 26.3)	5.7% more (1.2 more to 11.6 more)	MODERATE^a^	Compared with regular ICS regimen, the as needed ICS/FABA regimen probably has slightly higher risk of moderate to severe exacerbations
Severe exacerbations follow up: range 16 weeks to 44 weeks No. of participants: 870 (3 RCTs) [17, 26, 29]	RR 1.34 (1.02 to 1.75)	16.4%	22.0% (16.7 to 28.7)	5.6% more (0.3 more to 12.3 more)	MODERATE^b^	Compared with regular ICS regimen, the as needed ICS/FABA regimen probably has slightly higher risk of severe exacerbations
Time to first exacerbation follow up: range 12 weeks to 44 weeks No. of participants: 591 (3 RCTs) [17, 29, 30]	HR 1.295 (0.805 to 2.083)	11.1%	14.1% (9.0 to 21.8)	3.0% more (2.1 fewer to 10.6 more)	MODERATE^a^	Compared with regular ICS regimen, the as needed ICS/FABA regimen probably increases the HR for the time to first exacerbation
ICS compared to FABA for intermittent or mild persistent asthma						
Patient or population: intermittent or mild persistent asthma Setting: inpatients and outpatient Intervention: ICS Comparison: FABA						
Time to first exacerbation follow up: range 12 weeks to 44 weeks No. of participants: 536 (3 RCTs) [17, 29, 30]	HR 0.420 (0.274 to 0.644)	27.0%	12.4% (8.3 to 18.4)	14.6% fewer (18.7 fewer to 8.7 fewer)	MODERATE^a^	Compared with FABA regimen, regular ICS regimen probably reduces the HR for the time to first exacerbation

The risk in the intervention group (and its 95% confidence interval) is based on the assumed risk in the comparison group and the relative effect of the intervention (and its 95% CI)

GRADE Working Group grades of evidence:

High certainty: We are very confident that the true effect lies close to that of the estimate of the effect;

Moderate certainty: We are moderately confident in the effect estimate: The true effect is likely to be close to the estimate of the effect, but there is a possibility that it is substantially different;

Low certainty: Our confidence in the effect estimate is limited: The true effect may be substantially different from the estimate of the effect;

Very low certainty: We have very little confidence in the effect estimate: The true effect is likely to be substantially different from the estimate of effect

*No.* number, *CI* confidence interval, *RR* risk ratio, *HR* hazard ratio

^a^The results obtained for different diagnostic criteria of asthma and beclomethasone equivalent consumption

^b^The results obtained for different beclomethasone equivalent consumption

### Secondary outcomes

Compared with the FABA regimen, the ICS/FABA regimen showed a decrease in nocturnal awakenings (SMD = −0.29, 95%-CI = [−0.49, −0.09], *P* = 0.004, I^2^ = 8.7%, Table 5) and a marked improvement in FEV_1_% predicted (SMD = 0.77, 95%-CI = [0.11, 1.44], *P* < 0.001, I^2^ = 91.3%) but there was no significant difference in the percentage of asthma symptom-free days between these two regimens. The as-needed ICS/FABA regimen had a trend to reduce number of rescue medication required per day but this did not reach a statistical significance (SMD = −0.14, 95%-CI = [−0.29, 0.01], *P* = 0.076, I^2^ = 18.0%). In comparison with ICS regimen, the as-needed ICS/FABA regimen had a decreased percentage of asthma symptom-free days (SMD = −0.25, 95%-CI = [−0.37, −0.13], *P* < 0.001, I^2^ = 0.0%) and more rescue medication required per day (SMD = 0.19, 95%-CI = [0.08, 0.31], *P* = 0.001, I^2^ = 16.2%), but there was no statistical significance in nocturnal awakening times and improvement of FEV_1_% predicted between these two regimens.

**Table 5:** Summary of findings for secondary outcomes

Comparisons	Nocturnal awakenings (no.)						Symptom-free days (%)					
	No. of Studies	SMD	95% CI	*P* value	I^2^-squared (%)	Power	No. of Studies	SMD	95% CI	*P* value	I^2^-squared (%)	Power
ICS/FABA regimen vs. FABA regimen												
All	2 [17, 30]	−0.290	[−0.490, −0.090]	0.004	8.7	0.96	4 [17, 27, 29, 30]	0.083	[−0.074, 0.240]	0.303	22.9	0.94
Adult	1 [17]	−0.207	[−0.461, 0.047]	0.110	–	0.48	2 [17, 27]	0.068	[−0.147, 0.284]	0.534	0.0	0.16
Children and adolescent	1 [30]	−0.427	[−0.752, −0.102]	0.010	–	0.82	2 [29, 30]	0.099	[−0.131, 0.329]	0.398	71.0	0.94
ICS/FABA regimen vs. ICS regimen												
All	2 [17, 30]	−0.098	[−0.284, 0.087]	0.299	0.0	0.57	4 [17, 26, 29, 30]	−0.253	[−0.373, −0.132]	<0.001	0.0	0.81
Adult	1 [17]	−0.026	[−0.286, 0.234]	0.845	–	0.07	1 [17]	−0.160	[−0.421, 0.100]	0.228	–	0.32
Children and adolescent	1 [30]	−0.173	[−0.438, 0.091]	0.200	–	0.36	3 [26, 29, 30]	−0.278	[−0.414, −0.142]	<0.001	0.0	0.66
ICS regimen vs. FABA regimen												
All	2 [17, 30]	−0.261	[−0.465, −0.056]	0.012	0.0	0.96	3 [17, 29, 30]	0.275	[0.102, 0.448]	0.002	0.0	1.00
Adult	1 [17]	−0.212	[−0.475, 0.051]	0.115	–	0.47	1 [17]	0.249	[−0.015, 0.512]	0.064	–	0.56
Children and adolescent	1 [30]	−0.335	[−0.658, −0.011]	0.043	–	0.64	2 [29, 30]	0.295	[0.065, 0.525]	0.012	40.8	0.99
Comparisons	Rescue medication required per day						FEV_1_% of predicted value					
	No. of Studies	SMD	95% CI	*P* value	I^2^-squared (%)	Power	No. of Studies	SMD	95% CI	*P* value	I^2^-squared (%)	Power
ICS/FABA regimen vs. FABA regimen												
All	5 [17, 27–30]	−0.138	[−0.290, 0.014]	0.076	18.0	0.98	3 [17, 27, 29]	0.773	[0.105, 1.441]	0.023	91.3	1.00
Adult	3 [17, 27, 28]	−0.226	[−0.429, −0.023]	0.029	0.0	0.52	2 [17, 27]	0.405	[0.188, 0.623]	<0.001	0.0	0.94
Children and adolescent	2 [29, 30]	−0.025	[−0.254, 0.205]	0.834	46.0	0.34	1 [29]	1.429	[1.064, 1.794]	<0.001	–	1.00
ICS/FABA regimen vs. ICS regimen												
All	5 [17, 26, 28–30]	0.192	[0.075, 0.309]	0.001	16.2	1.00	2 [17, 29]	−0.061	[−0.629, 0.506]	0.832	86.3	0.96
Adult	2 [17, 28]	0.097	[−0.141, 0.335]	0.424	0.0	0.20	1 [17]	0.219	[−0.042, 0.480]	0.100	–	0.50
Children and adolescent	3 [26, 29, 30]	0.222	[0.088, 0.356]	0.001	49.5	0.91	1 [29]	−0.360	[−0.691, −0.030]	0.033	–	0.68
ICS regimen vs. FABA regimen												
All	4 [17, 28–30]	−0.152	[−0.318, 0.014]	0.073	0.0	0.87	2 [17, 29]	0.415	[−0.102, 0.933]	0.001	83.2	1.00
Adult	2 [17, 28]	−0.227	[−0.468, 0.014]	0.065	0.0	0.56	1 [17]	0.161	[−0.101, 0.424]	0.229	–	0.33
Children and adolescent	2 [29, 30]	−0.084	[−0.313, 0.014]	0.471	55.1	0.53	1 [29]	0.690	[0.356, 1.024]	<0.001	–	0.99

*no.* number, *SMD* standardized mean difference, *CI* confidence interval, *ICS* inhaled corticosteroids, *FABA* fast-onset-acting β_2_-agonist, *ICS/FABA* inhaled corticosteroids/fast-onset-acting β_2_-agonist

### Subgroup analysis

We also performed a subgroup analyses based on adults, and children or adolescents. In the adult subgroup, the as-needed ICS/FABA regimen had a significant decrease in moderate to severe exacerbations compared with the FABA regimen (RR = 0.24, 95%-CI = [0.10, 0.54], P = 0.001, I^2^ = 0.0%, Table 3) and the NNTB was 8 and the number of avoided events per 1000 was 120 (95%-CI = [73, 142], Table 5) but this failed to reach statistical difference in severe exacerbations (RR = 0.17, 95%-CI = [0.02, 1.35], *P* = 0.093, I^2^ = 0.0%). Furthermore, the as-needed ICS/FABA regimen significantly reduced rescue medication required per day (SMD = −0.23, 95% CI = [−0.43, −0.02], *P* = 0.029, I^2^ = 0.0%) and improved FEV_1_% predicted (SMD = 0.41, 95%-CI = [0.19, 0.62], *P* < 0.001, I^2^ = 0.0%) compared with the FABA regimen. In addition, we did not find any statistical difference in moderate to severe exacerbations (RR = 0.58, 95%-CI = [0.21, 1.57], *P* = 0.284), severe exacerbations (RR = 0.12, 95%-CI = [0.01, 2.38], *P* = 0.166), and other secondary outcomes (Table 5) between the ICS/FABA and regular ICS regimen.

In the children and adolescent subgroup, in comparison to the FABA regimen, the ICS/FABA regimen significantly reduced nocturnal awakenings times (SMD = −0.42, 95%-CI = [−0.75, −0.10], *P* = 0.010, Table 5) and improved FEV_1_% predicted (SMD = 1.42, 95%-CI = [1.06, 1.79], P < 0.001), but there was no statistical difference in moderate to severe exacerbations (RR = 0.76, 95%-CI = [0.53, 1.10], *P* = 0.147, I^2^ = 0.0%, Table 3) or severe exacerbations (RR = 0.72, 95%-CI = [0.49, 1.07], *P* = 0.107). When compared with the ICS regimen, the ICS/FABA regimen had higher of exacerbations (RR = 1.50, 95%-CI = [1.15, 1.96], *P* = 0.003, I^2^ = 18.4%) and severe exacerbations (RR = 1.40, 95%-CI = [1.07, 1.84], *P* = 0.014, I^2^ = 0.0%), a significant reduction in the percentage of asthma symptom-free days (SMD = −0.27, 95%-CI = [−0.41, −0.14], *P* < 0.001, I^2^ = 0.0%), more rescue medication required per day (SMD = 0.22, 95%-CI = [0.08, 0.35], *P* = 0.001, I^2^ = 49.5%) and reduced FEV_1_% predicted (SMD = −0.36, 95%-CI, [−0.69, −0.03], *P* = 0.033), but did not reach statistical significance on the nocturnal awakening times.

### Safety

There were 9.4% (*n* = 58), 15.8% (*n* = 50) and 7.7% (*n* = 43) of participates who withdrew from the studies in the ICS/FABA regimen, the FABA regimen and the ICS regimen, respectively, which indicated more dropouts in the FABA regimen when compared to the ICS/FABA and the daily ICS regimens in the children and adolescent subgroup (Additional file 1: Table S3). It reported eight serious adverse events (such as bacterial pneumonia and hemoptysis) in all included studies (Additional file 1: Table S4), which had no difference between regimens. In terms of the linear growth. The study by Martinez et al [29] indicated that, compared with the FABA group, children with the daily use of ICS regimen grew 1.1 cm (SD = 0.3) less (P < 0·0001) during the 44-week treatment period, but no significant growth effect was found in children with the as-needed ICS/FABA regimen (0.3 cm, SD = 0.2, *P* = 0.26). During the 16-week treatment interval, Fitzpatrick et al [26] found that children with the as-needed use of ICS/FABA regimen grew 0.2011 cm (SE = 0.2097, *P* = 0.3381) higher than children with the daily use of ICS regimen.

### Adherence of the daily use of ICS treatment

An additional meta-analysis was performed to pool the real-life adherence of the daily use of ICS treatment in patients with persistent asthma. The studies selection flow and characteristics of included studies were provided in the Methods and Results sections in the Additional file 1. The adherence of daily ICS regimen was calculated using the proportion of days covered (PDC) and defined as the total number of days with supply dispensed during the follow-up over the number of days of follow-up. Using a random-effects model, ICS adherence was 37.6% (95% CI = [33.1, 42.2], Additional file 1: Table S9).

## Discussion

To our best knowledge, this is the first proof-of-concept systematic review and meta-analysis to systematically explore the efficacy and safety of the ICS/FABA regimen in single inhaler as reliever therapy in intermittent and mild asthma. Our study included the six trials with 1300 patients and suggests that the as-needed use of ICS/FABA regimen significantly reduces exacerbations, including severe exacerbations, nocturnal awakening, prolongs time to first exacerbation and improves FEV_1_% predicted as compared to the as-needed FABA regimen, but it is inferior to the regular ICS regimen except for time to first exacerbation. The safety analysis indicated that the regular ICS regimen especially in a long-term treatment would lead to a small reduction in growth compared to either as-needed ICS/FABA or FABA regimens in the children and adolescent population. Our study shows that the ICS/FABA regimen in a single inhaler as a symptom-driven therapy would be a promising alternative regimen in management of intermittent or mild persistent asthma.

There are two important characteristics about the included RCTs for this meta-analysis. The first is the limited number of subjects involved in this meta-analysis, which resulted in under-powering with less than 0.80 to find statistical difference in some outcomes, especially in the sub-group meta-analysis, although most of the included studies calculated adequate power for their specific primary outcomes rather than ours in this study. The second is all included studies were completed in an ideal setting but not in real-world conditions [33], because all included subjects were requested to have an adherence of more than 75% before recruitment. In the real-world setting, the adherence of daily ICS was only 37.6% (95% CI = [33.1, 42.2]) in our additional meta-analysis. Therefore, the regular ICS regimen would not be more effective than the as-needed ICS/FABA strategy as poor adherence to ICS is correlated with asthma-related outcomes [34].

Despite effective pharmacological options for treating asthma, most patients fail to achieve good control in the real world. Non-adherence is common, with over-reliance on SABA and under-use of ICS frequently being observed. Therefore, there is a real need to consider new approaches to improve outcomes. One regimen that has attracted attention and controversy is single inhaler for maintenance and relief therapy (SMART). The SMART or single inhaler therapy (SiT) means that a single inhaler contains two drugs. One of these drugs acts quickly and termed the ‘reliever’, and the other one works much more slowly and is called the ‘preventer’. patients on SMART have one inhaler for use every day to control their underlying inflammation and also for symptom relief. The timely ICS use at the time of increased symptoms can improve asthma outcomes by reducing exacerbation risk [35]. In recent decades, evidence has demonstrated that using combined ICS and FABA as reliever medication can reduce the exacerbation rate [36–38] and have a lower total ICS exposure, without compromising outcomes against current best practice strategies using a fixed-dose ICS/LABA combination inhaler. But most of this evidence comes from moderate to severe asthma patients, who have a greater risk of exacerbations. Thus, GINA [1] recommended ICS/formoterol, a ICS/FABA inhaler, as reliever medication for moderate to severe asthma patients except for patients with intermittent and mild asthma. In our study, we extended this efficacy to the population with intermittent and mild asthma in significant improvements of nocturnal awakening times, FEV_1_% predicted, exacerbations and the time to first exacerbation compared the as-needed use of ICS/FABA regimen with the as-needed FABA regimen.

A possible concern with as-needed ICS/FABA is that inadequate anti-inflammatory treatment may be given to some patients, who would be at higher risk of exacerbations. Treatment with daily ICS at low doses decreases the risk of severe exacerbations and improves asthma control in patients with mild persistent asthma [39, 40]. Good adherence is needed for the efficiency of daily ICS therapy, but patients trend to use ICS intermittently and occasionally [15, 16]. Previous research has shown that nonadherence of ICS results in poor clinical control and increases school and work absenteeism, unscheduled health-care utilization, morbidity, and mortality [34, 41, 42]. We performed an additional meta-analysis in the adherence of the daily ICS therapy in a real-world setting in this study, and found the real-life adherence of daily use of ICS was 37.6% (95% CI = [33.1, 42.2]) with obvious heterogeneity between real-world RCTs and observational studies (Additional file 1: Table S9), while, it was more than 75% in our included studies significantly related to the reduced asthma exacerbations [43]. With regard to potential corticosteroid side effects, treatment with the as-needed ICS/FABA regimen was characterized by a lower average ICS dose and in children or adolescent population, by a higher linear growth than treatment with regular daily ICS regimen.

Based on the results of our study, the current recommendation from guidelines that regular ICS should be initiated only when patients use their SABA more than twice per week needs to be revisited, because the evidence that this approach works in real-life clinical practice is limited. The potential benefits of this approach were compromised by both low rates of ICS prescription in patients, even in the setting of poor control, and poor adherence by patients who were prescribed ICS regimen. Accordingly, the as-needed ICS/FABA regimen would be a promising alternative therapy, which might represent an effective, safe, and novel therapy for the treatment of intermittent and mild asthma. It may be particularly useful for selected patients who adhere poorly to their regular daily ICS regimen.

From a clinical point of view, the as-needed use of ICS/FABA regimen is a promising choice for the long-term management of intermittent and mild asthma. Exacerbations are major determinants of the direct cost of asthma, and preventing exacerbations is one of the key goals in asthma management [44]. In our study, compared with the as-needed use of FABA regimen, the as-needed use of ICS/FABA as monotherapy statistically reduced moderate to severe exacerbations and severe exacerbations. In addition, the number of patients that need to be treated for one of them to benefit from decreased moderate to severe exacerbations compared with the as-needed use of FABA regimen was 10 (the number of moderate to severe exacerbations that to be decreased from treating 1000 patients compared with the as-needed use of FABA regimen was 101 ((95%-CI = [51, 138])) and the number of patients that need to be treated for one of them to benefit from decreased severe exacerbations compared with the as-needed use of FABA regimen was 16 (the number of severe exacerbations that to be decreased from treating 1000 patients compared with the as-needed use of FABA regimen was 62 (95%-CI = [9, 96])). On the other hand, as we had mentioned above, good adherence is needed for the efficiency of daily ICS therapy, and there are some interventions to improve adherence to ICS may take many forms, including audiovisual reminders [45, 46], electronic monitoring [46], interactive voice response system via mobile phone [47], text message reminders [48] and parent education [49]. However, the magnitude of the improvements in adherence was generally not large (range from 4% to 20%) [50]. This improvement does not ensure the good adherence of ICS regimen (75%). Besides, the successful interventions to promote adherence were complex and multi-faceted and included combinations of counselling, education, more convenient care, self-monitoring, reinforcement, reminders, and other forms of additional attention or supervision [51, 52].

There are several limitations to this study that needs to be addressed. First, this study aimed to demonstrate the proof-of-concept whether the ICS/FABA in a single inhaler as reliever therapy in intermittent and mild persistent asthma was feasible in clinical practice, therefore two of included studies [29, 30] involved the use of the ICS and the FABA in separate inhalers but not in a single inhaler. Second, we included the limited number of studies that had inadequate power to find some difference in the subgroup analysis. Third, we used GetData Graph Digitizer to mine data and the Cochrane handbook recommended principals to deal with missing data, which would result in some potential impact on outcomes. Fourth, there was obvious heterogeneity in some outcomes such as the moderate to severe exacerbations, but it could be partly explained by different age groups. Fifth, all included RCTs were completed in an ideal condition with more than 75% of adherence rather than a real-world setting. To provide additional information in real-life adherence of regular ICS regimen, we pooled the rates of adherence across real-world RCTs and observational studies.

## Conclusions

In conclusion, our study suggests that the as-needed use of ICS/FABA regimen significantly reduces exacerbations, nocturnal awakening times, extend time to first exacerbation and improves FEV_1_% predicted compared to the as-needed FABA regimen, but it is inferior to the regular ICS regimen except for time to first exacerbation. With regard to potential corticosteroid side effects, it indicated that the regular ICS regimen especially in a long-term treatment would lead to grow less than either the as-needed ICS/FABA or FABA regimens in the children and adolescent population. This study displays that the ICS/FABA regimen in a single inhaler as a symptom-driven therapy would be a promising alternative regimen particularly for the very patients with intermittent or mild asthma who adhere poorly to their regular ICS regimen. However, further real-world RCTs are needed to confirm these findings.

## Additional files

Additional file 1: The online material of corticosteroid plus β2-agonist in a single inhaler as reliever therapy in intermittent and mild asthma: A proof-of-concept systematic review and meta-analysis. (DOCX 80 kb)

## Abbreviations

BDP: Beclomethasone dipropionate
CI: Confidence interval
FEV_1_: Forced expiratory volume in one second
HR: Hazard ratio
ICS: Inhaled corticosteroids
ICS/FABA: The combination corticosteroid/fast-onset-acting β_2_-agonist
LABA: Long-acting β_2_-agonists
NNT: The number needed to treat
NNTB: The number needed to treat for an additional beneficial outcome
NNTH: The number needed to treat for an additional harmful outcome
PDC: The proportion of days covered
RR: Risk ratio
SABA: Short-acting β_2_-agonist
SMD: The standardized mean difference
