# Supplementary material for: Corticosteroid plus β2-agonist in a single inhaler as reliever therapy in intermittent and mild asthma: a proof-of-concept systematic review and meta-analysis
Source: Respir Res. 2017 Dec 6;18:203. doi: 10.1186/s12931-017-0687-6 (PMC5718039; doi:10.1186/s12931-017-0687-6)
Supplement: Supplementary file 1 — The online material of corticosteroid plus β2-agonist in a single inhaler as reliever therapy in intermittent and mild asthma: A proof-of-concept systematic review and meta-analysis. (DOCX 80 kb) [file 12931_2017_687_MOESM1_ESM.docx]

**Online repository**

**Corticosteroid plus β_2_-agonist in a single inhaler** **as reliever therapy in intermittent and mild asthma: A proof-of-concept systematic review and meta-analysis**

Gang Wang, MD,^1, 2, 3*^, Xin Zhang, MD,^1, 2*^, Hong Ping Zhang, MD,^1, 2^, Lei Wang, MD,^1, 2^, De Ying Kang, PhD,^4^, Eric D. Bateman, MD, FRCP,^5^, Peter J. Barnes, FRS, FMedSci,^6^, Gang Wang, MD, PhD,^1, 2^

## Methods

**Digitizing**

GetData Graph Digitizer (DR MyCommerce, Inc.) is a program for digitizing graphs and plots. In this study, it was used to obtain original data from Kaplan–Meier curves, when data were not available. Digitizing included a four-step process. The first step is to open a Kaplan–Meier curves’ graph, and then, set the scale (coordinate system), digitize (automatically or manually), and the final step is to copy the data to the clipboard, or export to one TXT file for statistical analysis.

**Pooled adherence of the daily use of ICS in real-world clinical practice**

To explore the pooled adherence to ICS as maintenance treatment in a real-world setting an additional meta-analysis was conducted. The daily ICS adherence meta-analysis was from real-world studies that reported the adherence of regular daily ICS therapy. We used the words such as “real-world”, “real-life”, “pragmatic” and “naturalist” to confirm whether one study was a real-world design. The proportion of days covered (PDC) defined as the total number of days with supply dispensed during the follow-up over the number of days of follow-up to calculate the adherence of daily ICS therapy. The PubMed was searched until May 30, 2017 with no language and year limitation, and the further details about search strategies are shown in Table S5.

Two reviewers (XZ & GW) independently selected studies, extracted information and judged the evidence quality. As the twice daily use of ICS was the most prevalent prescription, we only included the adherence of twice or more time use of ICS. The Newcastle-Ottawa Scale (NOS)^(1)^ was used to judge the quality of observational studies. The moderate quality evidence of cohort studies was designed by meeting at least six criteria of the Newcastle-Ottawa Quality Assessment Scale (NOS) (selection of exposed and non-exposed cohorts (four items), comparability of cohorts (at least one star), and outcome assessment) and we downgraded the evidence a level for each criterion that was not met and upgraded the evidence by one level if the study had two stars for comparability. The two authors independently used a pragmatic-explanatory continuum indicator summary (PRECIS)^(2)^, an extension of the CONSORT statement^(3)^ and The Cochrane Collaboration’s tool for assessing risk of RCT bias^(4)^ to judge whether one RCT study met the criteria of pragmatic design and the quality. A random-effects model was used to synthesize the real-world daily ICS adherence if obvious heterogeneity was observed (I^2^ > 50%), otherwise we used a fixed effects model.

## Results

Our search strategy for the pooled real-world daily ICS adherence initially yielded 59 citations, two review authors independently selected studies and extracted data and finally 11 studies^(5-15)^ met the included criterion (Figure E1). There were 8 cohort studies and three RCTs including 51208 patients (Table S6). Using the Newcastle-Ottawa Scale (NOS), three cohort studies were judged high evidence quality, four of them were judged moderate evidence quality and the rest one was judged low evidence quality (Table S7). The quality of RCTs were generally good (Table S8). Using a random-effects model, the real-world daily ICS adherence was 37.6% (95% CI=[33.1, 42.2]), which is indicated in Table S9.

**References**

1. Wells. G, B. S, D. OC, J. P, V. W, M. L, et al. The Newcastle-Ottawa Scale (NOS) for assessing the quality of nonrandomised studies in meta-analyses. <http://www.ohri.ca/programs/clinical>˙epidemiology/oxford.asp. 2011.

2. Thorpe KE, Zwarenstein M, Oxman AD, Treweek S, Furberg CD, Altman DG, et al. A pragmatic-explanatory continuum indicator summary (PRECIS): a tool to help trial designers. CMAJ : Canadian Medical Association journal = journal de l'Association medicale canadienne. 2009;180(10):E47-57.

3. Zwarenstein M, Treweek S, Gagnier JJ, Altman DG, Tunis S, Haynes B, et al. Improving the reporting of pragmatic trials: an extension of the CONSORT statement. BMJ (Clinical research ed). 2008;337:a2390.

4. Higgins JPT, Green S (editors). Cochrane Handbook for Systematic Reviews of Interventions Version 5.1.0 [updated March 2011]. The Cochrane Collaboration, 2011. Available from [www.handbook.cochrane.org](file:///C:\Users\王\Desktop\www.handbook.cochrane.org).

5. Bukstein DA, Luskin AT, Bernstein A. "Real-world" effectiveness of daily controller medicine in children with mild persistent asthma. Annals of allergy, asthma & immunology : official publication of the American College of Allergy, Asthma, & Immunology. 2003;90(5):543-9.

6. Ducharme FM, Noya FJ, Allen-Ramey FC, Maiese EM, Gingras J, Blais L. Clinical effectiveness of inhaled corticosteroids versus montelukast in children with asthma: prescription patterns and patient adherence as key factors. Current medical research and opinion. 2012;28(1):111-9.

7. Blais L, Kettani FZ, Lemiere C, Beauchesne MF, Perreault S, Elftouh N, et al. Inhaled corticosteroids vs. leukotriene-receptor antagonists and asthma exacerbations in children. Respiratory medicine. 2011;105(6):846-55.

8. DiSantostefano RL, Boudiaf N, Stempel DA, Barnes NC, Greening AP. The frequency of, and adherence to, single maintenance and reliever therapy instructions in asthma: a descriptive analysis. NPJ primary care respiratory medicine. 2016;26:16038.

9. Engelkes M, Janssens HM, de Jongste JC, Sturkenboom MC, Verhamme KM. Prescription patterns, adherence and characteristics of non-adherence in children with asthma in primary care. Pediatric allergy and immunology : official publication of the European Society of Pediatric Allergy and Immunology. 2016;27(2):201-8.

10. Foster JM, Usherwood T, Smith L, Sawyer SM, Xuan W, Rand CS, et al. Inhaler reminders improve adherence with controller treatment in primary care patients with asthma. The Journal of allergy and clinical immunology. 2014;134(6):1260-8.e3.

11. Guenette L, Breton MC, Gregoire JP, Jobin MS, Bolduc Y, Boulet LP, et al. Effectiveness of an asthma integrated care program on asthma control and adherence to inhaled corticosteroids. The Journal of asthma : official journal of the Association for the Care of Asthma. 2015;52(6):638-45.

12. Wells KE, Peterson EL, Ahmedani BK, Williams LK. Real-world effects of once vs greater daily inhaled corticosteroid dosing on medication adherence. Annals of allergy, asthma & immunology : official publication of the American College of Allergy, Asthma, & Immunology. 2013;111(3):216-20.

13. Bender BG, Cvietusa PJ, Goodrich GK, Lowe R, Nuanes HA, Rand C, et al. Pragmatic trial of health care technologies to improve adherence to pediatric asthma treatment: a randomized clinical trial. JAMA pediatrics. 2015;169(4):317-23.

14. Blais L, Kettani FZ, Forget A, Beauchesne MF, Lemiere C, Ducharme FM. Assessing adherence to inhaled corticosteroids in asthma patients using an integrated measure based on primary and secondary adherence. European journal of clinical pharmacology. 2017;73(1):91-7.

15. Vollmer WM, Feldstein A, Smith DH, Dubanoski JP, Waterbury A, Schneider JL, et al. Use of health information technology to improve medication adherence. The American journal of managed care. 2011;17(12 Spec No.):Sp79-87.

| Table S1. Search strategy of RCTs involving the as-needed use of ICS/FABA therapy in this study. | |
| --- | --- |
| Asthma search filter |  |
| 1 | exp Asthma/ |
| 2 | asthma$.mp. |
| 3 | (antiasthma$ or anti-asthma$).mp. |
| 4 | Respiratory Sounds/ |
| 5 | wheez$.mp. |
| 6 | Bronchial Spasm/ |
| 7 | (bronch$ adj3 spasm$).mp. |
| 8 | bronchoconstrict$.mp. |
| 9 | exp Bronchoconstriction/ |
| 10 | (bronch$ adj3 constrict$).mp. |
| 11 | Bronchial Hyperreactivity/ |
| 12 | Respiratory Hypersensitivity/ |
| 13 | ((bronchial$ or respiratory or airway$ or lung$) adj3 (hypersensitiv$ or hyperreactiv$ or allerg$ or insufficiency)).mp. |
| 14 | ((dust or mite$) adj3 (allerg$ or hypersensitiv$)).mp. |
| 15 | Or/1-14 |
| RCTs search filter* |  |
| 1 | Randomized controlled trials as Topic/ |
| 2 | Randomized controlled trial/ |
| 3 | Random allocation/ |
| 4 | Double blind method/ |
| 5 | Single blind method/ |
| 6 | Clinical trial/ |
| 7 | exp Clinical Trials as Topic/ |
| 8 | Or/1-7 |
| 9 | (clinic$ adj trial$1).tw. |
| 10 | ((singl$ or doubl$ or treb$ or tripl$) adj (blind$3 or mask$3)).tw. |
| 11 | Placebos/ |
| 12 | Placebo$.tw. |
| 13 | Randomly allocated.tw. |
| 14 | (allocated adj2 random).tw. |
| 15 | Or/9-14 |
| 16 | 8 or 15 |
| 17 | Case report.tw. |
| 18 | Letter/ |
| 19 | Historical article/ |
| 20 | Review of reported cases.pt. |
| 21 | Review, multicase.pt. |
| 22 | Or/17-21 |
| 23 | 16 not 22 |
| ICS/FABA container search filter |  |
| 1 | exp Budesonide, Formoterol Fumarate Drug Combination/ |
| 2 | symbicort.mp. |
| 3 | "Budesonide/formoterol".mp. |
| 4 | "Beclometasone/formoterol".mp. |
| 5 | exp Mometasone Furoate, Formoterol Fumarate Drug Combination/ |
| 6 | dulera.mp. |
| 7 | zenhale.mp. |
| 8 | "mometasone/formoterol".mp. |
| 9 | Or/1-8 |
| ICS search filter |  |
| 1 | exp Adrenal Cortex Hormones/ |
| 2 | ICS.mp. |
| 3 | (beclomethasone* or beclometasone* or triamcinolone* or fluticasone* or budesonide* or betamethasone* or flunisolide* or ciclesonide* or mometasone*).mp. |
| 4 | (inhal* adj5 (steroid* or corticosteroid* or glucocorticoid*)).mp. |
| 5 | Or/1-4 |
| SABA search filter |  |
| 1 | bitolterol.mp. |
| 2 | carbuterol.mp. |
| 3 | fenoterol.mp. or exp Fenoterol/ |
| 4 | isoetharine.mp. or exp Isoetharine/ |
| 5 | bronkosol.mp. |
| 6 | bronkometer.mp. |
| 7 | pirbuterol.mp. |
| 8 | maxair.mp. |
| 9 | reproterol.mp. |
| 10 | rimiterol.mp. |
| 11 | salbutamol.mp. |
| 12 | exp Albuterol/ or albuterol.mp. |
| 13 | ventolin.mp. |
| 14 | salbutamolo.mp. |
| 15 | albuterol.mp. |
| 16 | "albuterol sulfate".mp. |
| 17 | "salbutamol sulfate".mp. |
| 18 | "AH 3365".mp. |
| 19 | levosalbutamol.mp. |
| 20 | levalbuterol.mp. or exp Levalbuterol/ |
| 21 | terbutaline.mp. or exp Terbutaline/ |
| 22 | brethine.mp. |
| 23 | bricanyl.mp. |
| 24 | brethaire.mp. |
| 25 | tulobuterol.mp. |
| 26 | "hokunalin tape".mp. |
| 27 | metaproterenol.mp. or exp Metaproterenol/ |
| 28 | SABA.mp. |
| 29 | Or/1-28 |
| Fast-onset LABA search filter |  |
| 1 | formoterol.mp. or exp Formoterol Fumarate/ |
| 2 | eformoterol.mp. |
| 3 | atimos.mp. |
| 4 | foradil.mp. |
| 5 | foradile.mp. |
| 6 | oxis.mp. |
| 7 | perforomist.mp. |
| 8 | LABA.mp. |
| 9 | Or/1-8 |
| Synthetic filter |  |
| 1 | Asthma search filter and RCTs search filter and (ICS/FABA container search filter or (ICS search filter and (SABA search filter or Fast-onset LABA search filter))) |
| *, Ovid library recommended MEDLINE Filters for Randomized Controlled Trials, available at http://resourcecenter.ovid.com/site/resources/expert_search/healthexp.html. We used different recommendations for different databases. | |
| The trade name of SABA was available at www.drugs.com. | |
| The search filters mentioned above were search strategy for MEDLINE and were adapted for use in the other databases. | |

| Table S2. Risk of bias of included randomized controlled trials involving the as-needed use of ICS/FABA therapy. | | | | | | | | | | | |
| --- | --- | --- | --- | --- | --- | --- | --- | --- | --- | --- | --- |
| Studies | Selection bias | |  | Performance bias |  | Detection bias |  | Attrition bias |  | Reporting bias | Other bias |
|  | Random sequence generation | Allocation concealment |  | Blinding of participants and personnel |  | Blinding of outcome assessment |  | Incomplete outcome data |  | Selective reporting |  |
| Papi *et al*, 2007^17^ | Low risk | Low risk |  | Low risk |  | Low risk |  | Low risk |  | Low risk | Unclear |
| Haahtela *et al*, 2006^27^ | Low risk | Unclear |  | Unclear |  | Unclear |  | Unclear |  | Unclear | Unclear |
| Lazarinis *et al*, 2014^28^ | Low risk | Low risk |  | Unclear |  | Unclear |  | Unclear |  | Low risk | Unclear |
| Martinez *et al*, 2011^29^ | Low risk | Low risk |  | Low risk |  | Low risk |  | Low risk |  | Low risk | Unclear |
| Papi *et al*, 2009^30^ | Unclear | Low risk |  | Low risk |  | Low risk |  | Low risk |  | Unclear | Unclear |
| Fitzpatrick *et al*, 2016^26^ | Low risk | Low risk |  | Low risk |  | Low risk |  | Low risk |  | Low risk | Low risk^*^ |
| *The following items were judged, (i) whether the cross-over design is suitable, (ii) whether there is a carry-over effect, (iii) whether only first period data are available, (iv) incorrect analysis and (v) comparability of results with those from parallel-group trials. | | | | | | | | | | | |

| Table S3. The risk ratio of withdrawals. | | | | | |
| --- | --- | --- | --- | --- | --- |
| Comparisons | Studies | Risk ratio | 95% CI | P value | I-squared (%) |
| ICS/FABA regimen vs FABA regimen |  |  |  |  |  |
| All | 5^17,27-30^ | 0.73 | [0.50, 1.06] | 0.10 | 40.0 |
| Adult | 3^17,27,28^ | 1.01 | [0.60, 1.72] | 0.96 | 27.9 |
| Children and adolescent | 2^29,30^ | 0.50 | [0.29, 0.87] | 0.01 | 0.0 |
| ICS/FABA regimen vs ICS regimen |  |  |  |  |  |
| All | 5^17,26,28-30^ | 1.24 | [0.86, 1.80] | 0.26 | 0.0 |
| Adult | 2^17,28^ | 1.02 | [0.59, 1.78] | 0.94 | 25.1 |
| Children and adolescent | 3^26,29,30^ | 1.43 | [0.87, 2.37] | 0.16 | 0.0 |
| ICS regimen vs FABA regimen |  |  |  |  |  |
| All | 4^17,28-30^ | 0.68 | [0.29, 1.63] | 0.39 | 60.4 |
| Adult | 2^17,28^ | 1.20 | [0.67, 2.14] | 0.55 | 42.8 |
| Children and adolescent | 2^29,30^ | 0.38 | [0.20, 0.72] | <0.01 | 0.0 |
| CI, confidence interval; ICS, inhaled corticosteroids; FABA, fast-onset-acting β_2_-agonist; ICS/FABA, inhaled corticosteroids/fast-onset-acting β_2_-agonist. | | | | | |

| Table S4. Serious adverse events reported in included randomized controlled trials. | | | |
| --- | --- | --- | --- |
| Studies | As-needed use of ICS/FABA regimen | As-needed use of FABA regimen | Daily use of ICS regimen |
| Papi *et al*, 2007^17^ | Hemoptysis (n=1) | No SAEs | Myocardial ischemia (n =1) |
| Haahtela *et al*, 2006^27^ | Migraine (n =1);  Traffic accident (tibial fracture) (n =1);  Mola hydatidosa* (n =1) | No SAEs | - |
| Lazarinis *et al*, 2014^28^ | No SAEs | No SAEs | No SAEs |
| Martinez *et al*, 2011^29^ | No SAEs | No SAEs | Viral meningitis (n =1) |
| Papi *et al*, 2009^30^ | No SAEs | SAE not described in details (n =1) | No SAEs |
| Fitzpatrick *et al*, 2016^26^ | No SAEs | - | Bacterial pneumonia (n =1) |
| SAE, serious adverse events; ICS, inhaled corticosteroids; FABA, fast-onset-acting β_2_-agonist; ICS/FABA, inhaled corticosteroids/fast-onset-acting β_2_-agonist. | | | |
| *, Mola hydatidosa was diagnosed at abortion. | | | |

| Table S5. PubMed search strategy of adherence of real-world daily ICS therapy. | |
| --- | --- |
| Field | Search strategy |
| Asthma | (asthma[MeSH Terms]) OR asthma[Title/Abstract] |
| ICS | (((adrenal cortex hormones[MeSH Terms]) OR ICS[Title/Abstract]) OR (((((((((beclomethasone*[Title/Abstract]) OR beclometasone*[Title/Abstract]) OR triamcinolone*[Title/Abstract]) OR fluticasone*[Title/Abstract]) OR budesonide*[Title/Abstract]) OR betamethasone*[Title/Abstract]) OR flunisolide*[Title/Abstract]) OR ciclesonide*[Title/Abstract]) OR mometasone*[Title/Abstract])) OR ((inhal*[Title/Abstract]) AND (((steroid*[Title/Abstract]) OR corticosteroid*[Title/Abstract]) OR glucocorticoid*[Title/Abstract])) |
| Adherence | (((((((((adheren*[Title/Abstract]) OR adhaeren*[Title/Abstract]) OR complian*[Title/Abstract]) OR co-operat*[Title/Abstract]) OR cooperat*[Title/Abstract]) OR nonadheren*[Title/Abstract]) OR nonadhaeren*[Title/Abstract]) OR noncomplian*[Title/Abstract]) OR noncooperat*[Title/Abstract]) OR concordance*[Title/Abstract] |
| Real-world | (((((real-world[Title/Abstract]) OR 'real world'[Title/Abstract]) OR real-life[Title/Abstract]) OR 'real life'[Title/Abstract]) OR pragmatic*[Title/Abstract]) OR naturalist*[Title/Abstract] |
| ICS, inhaled corticosteroid. | |

| Table S6. Characteristics of included studies reported real-world adherence of ICS therapy. | | | | | | | | | | |
| --- | --- | --- | --- | --- | --- | --- | --- | --- | --- | --- |
| Studies | Year | Countries | Study design | Duration (month) | Number of subjects (N) | Sex (% female) | Age group | Asthma severity | Adherence of daily ICS (%) | SD |
| Bukstein *et al*^5^ | 2003 | America | Cohort study | 12 | 52 | 17 (33) | Children and adolescent | Mild | 45.5 | 25.1 |
| Ducharme *et al*^6^ | 2012 | Canada | Cohort study | - | 169 | 97 (57) | Children and adolescent | Mild to moderate | 27 | 23.0 |
| Blais *et al*^7^ | 2011 | Canada | Cohort study | 11.7* | 26866 | 15012 (55.9) | Children and adolescent | Mild to severe | 14.4 | 11.8 |
| DiSantostefano *et al*^8^ | 2016 | United Kingdom | Cohort study | 12 | 14645 | 9148 (62.5) | Adult | - | 40 | 30 |
| Engelkes *et al*^9^ | 2016 | Netherlands | Cohort study | 30^*^ | 4000 | - | Children and adolescent | Mild to severe | 56 | 41.1 |
| Foster *et al*^10^ | 2014 | Australia | RCT | 6 | 43 | 27 (63) | Adolescent and adult | Mild to severe | 46 | 14.1 |
| Guenette *et al*^11^ | 2015 | Canada | Cohort study | 12 | 241 | 151 (62.7) | Adolescent and adult | Mild to severe | 30.1 | 32.9 |
| Wells *et al*^12^ | 2013 | America | Cohort study | 25* | 1081 | 656 (60.7) | Adolescent and adult | Mild to severe | 41 | 30 |
| Bender *et al*^13^ | 2015 | America | RCT | 24 | 447 | 174 (38.9) | Children and adolescent | - | 35.5 | 23.3 |
| Blais *et al*^14^ | 2017 | Canada | Cohort study | 12 | 404 | 209 (51.7) | Children, adolescent and adult | - | 43.5 | 30.1 |
| Vollmer *et al*^15^ | 2011 | America | RCT | 18 | 3260 | 2109 (64.7) | Adult | - | 38 | 32 |
| *, average follow-up time; N, number; RCT, randomized controlled trial. | | | | | | | | | | |

| Table S7. Risk of bias of cohort studies involving real-world adherence of ICS therapy. | | | | | | | | | |
| --- | --- | --- | --- | --- | --- | --- | --- | --- | --- |
| Studies | Selection | | | | Comparability of cohorts | Outcome | | | Evidence quality |
|  | Exposed cohort representative | Nonexposed cohort selection | Exposure ascertainment | Outcome not present at start |  | Assessment | Follow-up length | Follow-up adequacy |  |
| Bukstein *et al*, 2003^5^ | * | * | * | * | ** | * | * | * | High |
| Ducharme *et al*, 2012^6^ | * | * | * | * | ** | * | - | * | High |
| Blais *et al*, 2011^7^ | * | * | * | * | ** | * | * | * | High |
| DiSantostefano *et al*, 2016^8^ | * | * | * | * | - | * | * | * | Low |
| Engelkes *et al*, 2016^9^ | * | * | * | * | * | * | * | - | Moderate |
| Guenette *et al*, 2015^11^ | * | * | * | * | * | * | * | * | Moderate |
| Wells *et al*, 2013^12^ | * | * | * | * | * | * | * | * | Moderate |
| Blais *et al*, 2017^14^ | * | * | * | * | * | * | * | * | Moderate |
| Newcastle-Ottawa Quality Assessment Scale: 1 star (*) for meeting each criterion, except comparability (design or analysis) can have 2 stars. For comparability in this review: 1 star if controlled for asthma severity, 2 stars if also controlled for other important variables, e.g., age, daily dose of ICS. | | | | | | | | | |
| Moderate quality evidence: met criteria for selection (4 items), comparability (1 star; upgraded for 2 stars), and outcome assessment. Downgrading due to design limitation or lack of information in report. | | | | | | | | | |

| Table S8. Risk bias randomized controlled trials involving real-world adherence of ICS therapy. | | | | | | | | | | | | |
| --- | --- | --- | --- | --- | --- | --- | --- | --- | --- | --- | --- | --- |
| Studies | Selection bias | |  | Performance bias |  | Detection bias |  | Attrition bias |  | Reporting bias |  | Other bias |
|  | Random sequence generation | Allocation concealment |  | Blinding of participants and personnel |  | Blinding of outcome assessment |  | Incomplete outcome data |  | Selective reporting |  |  |
| Foster *et al*, 2014^10^ | Low risk | low risk |  | low risk |  | low risk |  | low risk |  | low risk |  | unclear |
| Bender *et al*, 2015^13^ | unclear | low risk |  | low risk |  | low risk |  | low risk |  | low risk |  | unclear |
| Vollmer *et al*, 2011^15^ | unclear | unclear |  | unclear |  | low risk |  | low risk |  | low risk |  | unclear |

| Table S9. Pooled adherence of the daily ICS therapy in real-world clinical practice. | | | | |
| --- | --- | --- | --- | --- |
| Included studies | Studies | Adherence (%) | 95% CI | I-squared (%) |
| Mild to moderate | 2^5,6^ | 35.3 | [17.2, 53.3] | 82.7 |
| Mild to severe | 7^5-7,9-12^ | 36.8 | [21.4, 52.1] | 99.9 |
| Pragmatic RCTs | 3^10,13,15^ | 37.7 | [35.6, 39.7] | 11.2 |
| Observational studies | 8^5-9,12,14^ | 37.1 | [23.1, 51.1] | 99.9 |
| Duration > 12 month | 8^5,8,9,11-15^ | 41.1 | [35.3, 47.0] | 98.2 |
| Total | 11^5-15^ | 37.6 | [33.1, 42.2] | 99.8 |
| N, number, CI, confidence interval. | | | | |
